# Effect of Locally Delivered Melatonin as an Adjunct to Nonsurgical Therapy on GCF Antioxidant Capacity and MMP-9 in Stage II Periodontitis Patients: A Randomized Controlled Clinical Trial

**DOI:** 10.1155/2021/8840167

**Published:** 2021-02-05

**Authors:** Enji Ahmed, Olfat G. Shaker, Nermin Yussif, Dalia M. Ghalwash

**Affiliations:** ^1^Oral Medicine and Periodontology Department, Faculty of Dentistry, Cairo University, Giza, Egypt; ^2^Department of Oral Medicine and Periodontology, Faculty of Dentistry, The British University in Egypt, El Sherouk City, Egypt; ^3^Medical Biochemistry and Molecular Biology Department, Faculty of Medicine, Cairo University, Giza, Egypt

## Abstract

**Objectives:**

Periodontitis is characterized by inflammatory destruction of periodontal tissue, loss of attachment, and bone resorption. The increase in reactive oxygen species (ROS) is responsible for the oxidative damage occurring in periodontal tissues. Melatonin has important immunomodulatory, anti-inflammatory, and powerful antioxidant functions. The current study was carried out to evaluate the effect of topical melatonin gel as an adjunct to nonsurgical periodontal therapy.

**Methods:**

This split-mouth randomized controlled clinical trial was performed on 24 patients with grade II periodontitis. Two sites in each patient were randomly assigned; test sites were treated by nonsurgical therapy followed by intrapocket application of 5% melatonin gel. Control sites were treated by nonsurgical therapy followed by intrapocket application of placebo gel. Both the melatonin and placebo gel were applied weekly once for four weeks. Assessment of clinical parameters (PD and CAL) was done at baseline and 3 months after therapy. Total antioxidative capacity (TAC) and matrix metalloproteinase-9 (MMP-9) levels in GCF were also evaluated utilizing commercially available enzyme-linked immunosorbent assay kits (ELISA) at baseline and 3 months after therapy.

**Results:**

Treatment with topical melatonin was associated with a reduction in periodontal inflammation reflected as an improvement in the clinical periodontal parameters. Melatonin-treated sites showed a more statistically significant percent reduction in PD and more statistically significant percent gain in CAL than the control site. Additionally, a significant increase in TAC and a significant decrease in MMP-9 levels in GCF were found in melatonin-treated sites in comparison to control sites.

**Conclusions:**

The adjunctive use of topical melatonin gel with nonsurgical periodontal therapy has potent anti-inflammatory and antioxidant activity in the treatment of grade II periodontitis patients.

## 1. Background

Periodontal disease is an oral inflammatory process that is typically initiated by bacterial infection stimulating the host response to produce various inflammatory mediators in high levels, which are also involved in the initiation and progression of periodontal disease [1]. Such mediators generate a cascade reaction that eventually leads to the irreversible degradation of connective tissues and bone, with subsequent loss of periodontal attachment [2].

Periodontal tissue infiltration by polymorphonuclear leukocytes (PMNs) and macrophages and consequent phagocytosis features a burst of O_2_ consumption producing higher concentrations of reactive oxygen species (ROS) that exerts a cytotoxic effect on periodontal tissues [3]. Various studies have demonstrated that oxidative stress induced by PMNs can be the primary etiology for the damage of periodontal tissue in periodontal disease [4]. It is worth highlighting that patients with periodontitis have often revealed higher levels of biomarkers indicating ROS-induced tissue damage than that observed in matched controls [5].

Matrix metalloproteinases (MMPs) are crucial proteases involved in periodontal tissue destruction. MMP-9 is a type IV collagenase that originates from PMNs, fibroblasts, macrophages, epithelial cells, and pathogenic bacteria [6]. It is one of the main proteases engaged in destructive periodontal disease and controls a number of functions related to inflammation. MMP-9 levels are heightened during periodontal inflammation [7].

Melatonin is a natural compound produced by the pineal gland, bone marrow, retina, and the immune system with a primary function of regulating the circadian rhythm [8]. It also plays an anti-inflammatory, immunomodulatory, and antioncotic role. Melatonin inhibits invasion, endometriosis, and fibrosis whilst boosting embryogenesis and wound healing by regulating the expression of ROS, MMPs, and growth factors [9].

Melatonin has been studied extensively and has shown to reduce oxidative stress through several different mechanisms. On the one hand, melatonin acts as an active scavenger of exogenous and endogenous ROS. On the other hand, melatonin also stimulates some vital antioxidant enzymes, namely, superoxide dismutase, glutathione reductase, glutathione peroxidase, and vitamin C [10].

Of particular importance, melatonin was found to influence bone regeneration and fibroblast activation by stimulating osteoblast differentiation and bone formation. Moreover, melatonin promotes type I collagen fibers synthesis [11]. Additionally, melatonin treatment was reported to stimulate osteoblast proliferation, differentiation, and activation while inhibiting bone resorption [11].

In addition to the downregulatory effect of melatonin on the expression of proinflammatory mediators such as interleukin-6, prostaglandins, *C*-reactive protein, and tumor necrosis factor-alpha, it also downregulates the receptor activator of nuclear factor kappa beta ligand (RANKL)/osteoprotegerin (OPG) ratios to decrease periodontal inflammation [12].

Several studies were conducted evaluating the melatonin effect on periodontal disease but as oral supplementation or in the topical forms other than the gel form used in the present study. Melatonin treatment has been described to decrease periodontitis in diabetic rats [13]. Moreover, topical melatonin application in patients with diabetes was related to a decrease in salivary RANKL concentrations and rise in salivary OPG concentrations, indicating that melatonin has the potential of reducing bone loss, improving bone quality, and hindering the progression of periodontal destruction [12, 14, 15]. Furthermore, local melatonin administration in rats produced a significant reduction of bone resorption [16]. According to the mentioned studies, it can be postulated that combining topical administration of melatonin with conventional periodontal therapy could enhance the treatment outcomes [17].

Consequently, the current study was designed to evaluate the effect of topical melatonin gel as an adjunct to nonsurgical therapy versus nonsurgical therapy alone on the clinical parameters (PI, GI, PD, and CAL) as well as total antioxidative capacity (TAC) and MMP-9 in GCF of stage II periodontitis patient.

## 2. Study Population and Methodology

### 2.1. Study Design

This split-mouth randomized controlled clinical trial was carried out in Periodontology clinic, Faculty of Dentistry, Cairo University.

### 2.2. Patients' Selection

**Figure 1:**
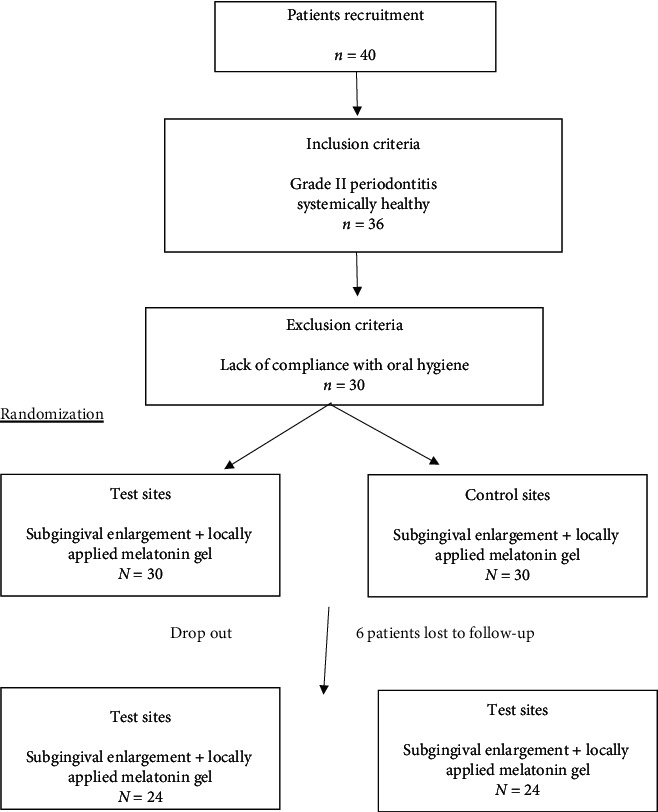
Flowchart of the study.

This study was conducted on a total of 30 patients (6 patients did not show up at follow-up) ending in 24 patients (17 females and 7 males with age range 32–55) having stage II periodontitis. Patients were recruited from the Periodontology Clinic, Faculty of Dentistry, Cairo University, and informed about the follow-up visits needed (Figure 1). They also instructed to avoid any medication that may interfere with the trial during the study.

The aim of the current study was to evaluate the effect of topical melatonin gel as an adjunct to nonsurgical periodontal therapy on the clinical parameters (pocket depth, PD; clinical attachment loss, CAL; plaque index, PI; and gingival index, GI) as well as TAC and MMP-9 level in GCF of stage II periodontitis patient. Total sample size of 24 patients was sufficient to detect the medium effect size (*f* = 0.3) estimated by Cutando et al. with power 85% and 5% significance level [18]. This number had to be increased to 30 patients to overcome losses during follow-up done by G power.

### 2.3. Eligibility Criteria

Patients eligible for the trial must be complied with all the following: patients were selected to be systemically free according to the modified Cornell Medical Index. Patients with stage II periodontitis was diagnosed as having interdental CAL is detectable at ≥2 nonadjacent teeth, or buccal or oral CAL ≥3 mm with pocketing >3 mm is detectable at ≥2 teeth and CAL 3-4 mm and maximum probing depth ≤5 mm [19]. Exclusion criteria include smokers, pregnant and lactating females, patients received any type of periodontal treatment in the past 6 months prior to examination, patients who used antibiotic or anti-inflammatory drugs or antioxidants within the 6 months preceding the beginning of the study, and patients working in night shifts or received any drug that known to alter melatonin levels (e.g., for sleeping disorders).

## 3. Ethical Procedures

Each patient was informed about the detailed procedure, the side effects of biopsy taking, and follow-up appointments, if needed. Each subject signed an informed written consent form. The patients consent to photography, charting, as well as radiographic examination to be performed, provided that their identity is not revealed and understood why it was necessary to accomplish these procedures. The Research Ethics Committee of Faculty of Dentistry, Cairo University, revised and approved this research on 24/12/2017 with the number (17-12-27).

### 3.1. Preoperative Evaluation

Patients eligible for the study were screened by a comprehensive periodontal examination, and full periodontal charts were obtained. Periapical radiographs were taken for periodontitis patients, at sites of attachment loss for diagnosis of the case.

After proper examination and diagnosis, full mouth supra- and subgingival scaling and root planning were performed in all patients over 2 weeks using scalers and Gracey Curettes from Hu-Friedy, and all patients received an oral hygiene instruction.

### 3.2. Intervention

Two sites in each patient were randomly assigned using random allocation computer software to receive one of the following treatment modalities as follows: the test site was treated by nonsurgical therapy followed by intrapocket application of 5% melatonin gel using a plastic disposable syringe with plastic flexible tip preloaded to deliver the melatonin inside the pocket. The control site was treated by nonsurgical therapy followed by intrapocket application of placebo gel using a plastic disposable syringe with plastic flexible tip (manufactured by SUNG SHIM Medical CO., LTD, made in Korea insulin syringe, 1 ml with gauge 29G). Both the melatonin and placebo gel were applied weekly once for four weeks.

#### 3.2.1. Melatonin Oral Gel Preparation

Pure melatonin was purchased from Bulk Supplements (USA) Company and prepared as an oral gel 5% by El Ezaby Pharmacy. The used base was 1% carboxymethyl cellulose in distilled water. The placebo gel was also prepared by El Ezaby Pharmacy and consisted of 1% carboxymethyl cellulose in distilled water alone.

### 3.3. Allocation

The sequence was generated through computer-generated random numbers with an allocation ratio of 1 : 1. The melatonin and placebo gels were identical in appearance and were placed in prepacked identical syringes (those used for insulin injection) which were numbered for each patient according to the randomization schedule. Sequentially numbered containers for concealment were done by the coinvestigator.

## 4. Implementation

The main investigator was responsible for the enrollment of participants, assigning participants to interventions, and performing treatment. The coinvestigators were responsible for generating the allocation sequence. The main investigator was responsible for the assessment of outcomes.

### 4.1. Blinding

The current study was triple blinded. Blinding was performed to the patients, the evaluator of outcomes, and the statistician.

### 4.2. Strategies to Improve Adherence to Intervention Protocol

Printed follow-up schedule cards with reminder calls or messages were utilized to improve adherence.

## 5. Outcomes

### 5.1. Clinical Parameters

The following clinical parameters were assessed by the main investigator at baseline and 3 months after periodontal treatment:

1. Plaque Index: the amount of dental plaque was assessed by the Plaque Index in order to monitor oral hygiene performance by the patient [20].
2. Gingival Index (GI): the gingival status was evaluated using the Gingival Index to evaluate the presence or absence of gingival inflammation [21].
3. Probing pocket depth (PD): the distance between the gingival margin and the deepest part of the pocket was measured using William's graduated periodontal probe.
4. Clinical attachment level (CAL): clinical attachment loss was measured from the cemento-enamel junction to the deepest part of the pocket using William's graduated periodontal probe.

### 5.2. Biochemical Analysis

Evaluation of changes in the GCF level of TAC and MMP-9 was done using enzyme-linked immunosorbent assay (ELISA). The unit of measurement is micromole (*μ*mol/L) in case of TAC and picogram (pg/*µ*l) in case of MMP-9.

## 6. Collection of Gingival Crevicular Fluid (GCF) Samples

The GCF samples were collected from the sites with the deepest PD and radiographic evidence of bone loss. The sample was collected at baseline and 3 months following periodontal treatment. The GCF samples were obtained as follows: after proper isolation of the sample site with cotton rolls (to prevent contamination with saliva), the tooth was air-dried, and a sterile filter paper strip (1.5 × 20 mm) was inserted into the sulci/pockets until minimal resistance was felt and left in the place for 1 minute. Strips contaminated by blood or saliva were excluded. After GCF collection, the strips were placed immediately in sterile Eppendorf tubes. The tubes were stored in liquid nitrogen (−80°C) until biochemical analysis [22].

## 7. Determination of TAC in GCF

The determination of the antioxidative capacity was performed using a kit provided by Immundiagnostik, Germany, by the reaction of antioxidants in the sample with a defined amount of exogenously provided (H_2_O_2_), and the antioxidants in the sample eliminated a certain amount of the provided hydrogen peroxide. The residual H_2_O_2_ was determined photometrically by an enzymatic reaction which involved the conversion of tetramethylbenzidine (TMB) to a colored product. After addition of a stop solution, the samples were measured at 450 nm in a microtiter plate reader. The quantification was performed by the delivered calibrator.

## 8. Determination of MMP-9 in GCF

Determination of MMP-9 levels in GCF was done using human MMP-9 ELISA kit produced by Affymetrix eBioscience. This assay is a quantitative sandwich enzyme immunoassay technique. A monoclonal antibody specific for MMP-9 has been precoated onto a microplate. Samples were poured into the wells, and any MMP-9 present was conjugated with the immobilized antibody. The unconjugated remnants were removed by washing. The enzyme-linked monoclonal antibody specific for MMP-9 was added to all wells. A substrate solution was inserted into the wells after washing to remove any unconjugated antibody-enzyme reagent. Color appeared in proportion to the amount of MMP-9 conjugated in the first step. The color development was stopped, and the intensity of the color was measured by ELISA reader.

## 9. Statistical Analysis

Kolmogorov–Smirnov and Shapiro–Wilk tests were used to check if numerical data had a normal distribution or not. PD, CAL, TAC, and MMP-9 data showed parametric distribution while GI and PI data showed nonparametric distribution. Numerical data were presented as mean, median, standard deviation (SD), minimum, maximum, and 95% confidence interval (95% CI) values. For parametric data, a paired *t*-test was used to compare between test and control sites as well as to study the changes after treatment.

For nonparametric data, the Wilcoxon signed-rank test was used to compare between test and control sites as well as to study the changes after treatment. The Kruskal–Wallis test was used to compare between TAC and MMP-9 levels of the two sites. Mann–Whitney *U* test with Bonferroni's adjustment was used for pairwise comparisons when the Kruskal–Wallis test is significant. The significance level was set at *P* ≤ 0.05. Statistical analysis was performed with IBM® SPSS® Statistics Version 20 for Windows.

## 10. Results

The present study included a total of 30 patients (6 patients did not show up at follow-up) ending in 24 patients (12 females and 10 males with age range 32–55) having stage II periodontitis. In each of the 24 patients, two sites were randomly assigned to receive one of the intervention protocols (nonsurgical therapy and topical application of melatonin gel versus nonsurgical therapy and topical application of placebo gel). No adverse effects were reported by the patients who received 5% of melatonin oral gel or placebo gel.

### 10.1. Clinical Parameters

The results of the clinical parameters at baseline and 3 months after treatment are shown in Tables 1 and 2. Regarding PI, both sites (test and control) showed statistically significant reduction in plaque scores 3 months after treatment. At the end of the follow-up period, there was no statistically significant difference between mean PI in both sites. The result applying to GI in both sites showed a statistically significant reduction in GI scores 3 months after treatment, whereas there was no statistically significant difference between both sites at the end of the study.

Regarding PD and CAL, both sites showed statistically significant reduction in PD and gain in CAL 3 months after treatment; however, test site showed more statistically significant percent reduction in PD and more statistically significant percent gain in CAL than the control site.

### 10.2. Biochemical Analysis

#### 10.2.1. Total Antioxidant Capacity (TAC%)

At the test site, mean ± SD of TAC at baseline was 284.5 ± 33.2 (*μ*mol/L) that increased to 584.4 ± 64.1 (*μ*mol/L) after treatment. Mean difference at baseline and 3 months after treatment was 299.9 with a 95% confidence interval (CI) (254.6–345.1). This difference was statistically significant (*P* < 0.001).

At the control site, mean ± SD of TAC at baseline was 283.2 ± 30.9 (*μ*mol/L) that increased to 437.3 ± 60.4 (*μ*mol/L) 3 months posttreatment. Mean difference at baseline and after three months was 154.1 with 95% CI (114.4–193.8). This difference was statistically significant (*P* < 0.001).

**Figure 2:**
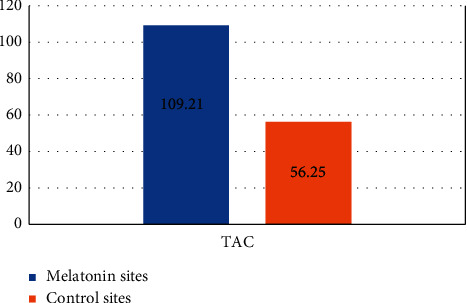
Bar chart of TAC percent change in both sites.

Although both sites showed a statistically significant increase in TAC, the test site showed a more statistically significant % increase in TAC (109.21 ± 41.79%) than the control site with a % increase in TAC (56.25 ± 27.64%) with *P* < 0.001 (Figure 2).

#### 10.2.2. Matrix Metalloproteinase-9

At the test site, mean ± SD of MMP-9 at baseline was 77.71 ± 2.86 (pg/*µ*L) that decreased to 31.20 ± 2.3 (pg/*µ*L) 3 months after treatment. There was a statistically significant reduction in the MMP-9 level with *P* < 0.001. At control site, mean ± SD of MMP-9 at baseline was 77.53 ± 3.4 (pg/*µ*L) and then decreased to 47.1 ± 1.8 (pg/*µ*L) 3 months after treatment. There was a statistically significant reduction in the MMP-9 level with *P* < 0.001.

**Figure 3:**
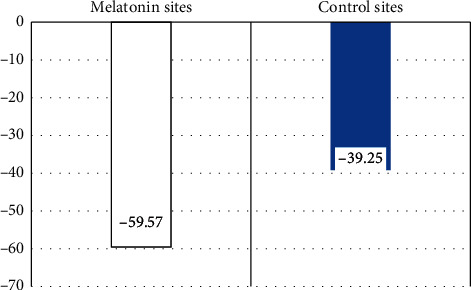
Bar chart of MMP-9 percent change in both sites.

Although both sites showed a statistically significant reduction in MMP-9, test site showed more statistically significant % reduction in MMP-9 (−59.57 ± 14.42%) than the control site with % reduction in MMP-9 of −39.25 ± 16.46% with *P* < 0.001 (Figure 3).

## 11. Discussion

Periodontal disease is characterized by inflammation-induced damage of periodontal tissue, loss of attachment, and alveolar bone loss. It is well understood that the increase in ROS produced by PMNs or other cells such as fibroblasts in periodontal disease stands behind the oxidative damage occurring in periodontal tissues which could be also associated with a reduction in the antioxidant defense mechanism [23]. When endogenous antioxidants are unable to lessen the damaging effects of ROS efficiently, tissue damage can result. The host capability to scavenge ROS is considered as a vital defensive mechanism to counteract the ROS-related periodontal tissue damage [5].

Melatonin is a pleiotropic multitasking molecule; among its versatile functions, it has an immunomodulatory and anti-inflammatory effects, and it is a remarkable free radical scavenger at the same time [24]; such functions are of paramount significance from a periodontal perspective as per augmenting the host defense and restoring the balance between the antioxidant and the pro-oxidant systems, thus reducing the oxidative damage and destruction of periodontal tissue. The present study was hence planned to estimate the effect of locally delivered melatonin gel as an adjunct to nonsurgical periodontal therapy on the clinical parameters (PD, CAL, PI, and GI) as well as TAC and MMP-9 levels in GCF of stage II periodontitis patient. In the present study, melatonin was applied in a gel form inside the pocket to increase the bioadhesion properties of the material and thus prolong its biological effects.

Results of the present study emphasize the favorable benefits of the local delivery of melatonin gel on periodontal disease reflected by the favorable effects obtained concerning the clinical parameters including PI, GI, PD, and CAL. Comparisons in all parameters pre and posttreatment with melatonin yielded statistically significant results. Moreover, melatonin-treated sites showed more statistically significant percent reduction in PD and more statistically significant percent gain in CAL than the control site. These results are in accordance with the study of Montero et al. [24] that reported that the topical application of melatonin has a number of positive effects on periodontal health, and it resulted in significant improvement of clinical parameters as the Gingival Index and pocket depth and also reported improved bone formation and osteoblast differentiation subsequent to topical application of melatonin.

An adequate balance between the host tissue production of ROS and TAC plays a vital role in periodontal tissue homeostasis leading to the prevention of tissue destruction upon immune system stimulation by periodontal pathogenic challenge [25]. The potent antioxidant effect of melatonin was confirmed in our study as per the statistically significant increase in TAC that was evident 3 months after starting the local delivery of melatonin. Moreover, test sites showed a more statistically significant percent increase in TAC than the control site.

This was in line with previous studies which observed that the considerable oxidative stress in periodontitis patients was significantly reduced by melatonin administration and declared that not only melatonin but also several endogenously generated metabolites of melatonin function as free radical scavenger [5]. Similarly, melatonin application was reported to protect the periodontal tissues against ROS injury resulting from inflammatory reactions by reducing the elevated oxidative stress levels in these patients and that it may reduce alveolar bone loss [26]. Moreover, melatonin supplementation adjunctive to nonsurgical periodontal therapy significantly increased the serum levels of TAC compared with the baseline [27].

Degradation of collagen fibers occurring in periodontal disease is a vital step in periodontal attachment loss. This degradation is achieved by MMPs released by the resident periodontal cells in response to the inflammatory stimuli. MMP-9 belongs to the collagenases family and is known to contribute significantly to connective tissue destruction [28]. MMP-9 levels were reported to be increased during periodontal inflammation [7]. And a significant decline in MMP-9 levels following periodontal therapy has also been observed [6].

In this study, combining locally delivered melatonin with nonsurgical periodontal therapy resulted in a significant decrease in GCF MMP-9 levels and showed more significant percent reduction in MMP-9 than the control site. To the best of our knowledge, this is the first study to assess the effect of locally delivered melatonin on GCF MMP-9 levels in periodontitis and so it is difficult to exactly correlate our results. However, the effect of melatonin on MMP-9 was investigated in association with other diseases and conditions where melatonin was reported to cause significant inhibition of the MMP-9 activity in both a dose- and time-dependent manner [29]. Additionally, melatonin was found to suppress MMP-9 expression at the protein and mRNA levels and to inhibit the enzymatic activity of MMP-9 [30]. The inhibition of the MMP-9 activity by melatonin was suggested to be due to the suppression of TNF-*α* release by melatonin [9].

## 12. Conclusion

The findings of the present study revealed a significant increase in TAC after intrapocket application of melatonin gel reflecting its powerful antioxidant potential together with a significant reduction of MMP-9, the marker of connective tissue degradation. With the above background, it can be concluded that the adjunctive effects of locally delivered melatonin gel with nonsurgical periodontal therapy have a positive effect on inflammatory and antioxidant parameters in periodontitis patients conferring a new aspect to the management of periodontitis. Thus, melatonin with the validated immunomodulatory, anti-inflammatory, and powerful antioxidant functions can be considered as a novel addition to the standard of care procedures for periodontal therapy to enhance treatment outcome.

## Figures and Tables

**Table 1:** Mean and standard deviation values for inter- and intragroup comparisons in all clinical variables between test and control sites.

	Test site (melatonin gel), *n* = 24	Control site (placebo gel), *n* = 24	*P* value
*Plaque Index (PI)*			
Baseline	2 ± 0.7	1.96 ± 0.6	0.83
Posttreatment	0.7 ± 0.6	0.7 ± 0.6	0.83
*Pvalue*	<0.0001^*∗*^	<0.0001^*∗*^	

*Gingival Index (GI)*			
Baseline	2.5 ± 0.5	2.4 ± 0.5	0.5
Posttreatment	0.6 ± 0.5	0.7 ± 0.5	0.5
*Pvalue*	<0.001^*∗*^	<0.001^*∗*^	

*Probing depth (PD)*			
Baseline	4.3 ± 0.8	4.0 ± 0.6	0.1485
Posttreatment	2.9 ± 0.7	3.1 ± 0.7	0.3275
*Pvalue*	<0.001^*∗*^	<0.001^*∗*^	

*Clinical attachment level (CAL)*			
Baseline	4.7 ± 0.9	4.3 ± 0.6	0.07
Posttreatment	3.5 ± 0.6	3.7 ± 0.6	0.254
*Pvalue*	<0.001^*∗*^	<0.001^*∗*^	

**Table 2:** Mean and standard deviation values for comparing % changes in all clinical variables among test and control sites.

Clinical parameters	Test site (melatonin gel), *n* = 24	Control site (placebo gel), *n* = 24	*P* value
*Plaque Index (PI)*			
Baseline	2 ± 0.7	1.96 ± 0.6	0.83
Posttreatment	0.7 ± 0.6	0.7 ± 0.6	0.83
% change	−65%	−64.29%	<0.001^*∗*^

*Gingival Index (GI)*			
Baseline	2.5 ± 0.5	2.4 ± 0.5	0.5
Posttreatment	0.6 ± 0.5	0.7 ± 0.5	0.5
% change	−80%	−70.83%	<0.001^*∗*^

*Probing depth (PD)*			
Baseline	4.3 ± 0.8	4.0 ± 0.6	0.1485
Posttreatment	2.9 ± 0.7	3.1 ± 0.7	0.3275
% change	−32.56%	−22.5%	<0.001^*∗*^

*Clinical attachment level (CAL)*			
Baseline	4.7 ± 0.9	4.3 ± 0.6	0.07
Posttreatment	3.5 ± 0.6	3.7 ± 0.6	0.254
% change	−25.53%	−13.95%	<0.001^*∗*^

## Data Availability

The data that support the findings of this study are available from faulty of Dentistry, Cairo University, but restrictions apply to the availability of these data, which were used under license for the current study and so are not publicly available. Data are however available from the authors upon reasonable request and with permission of faculty of Dentistry, Cairo University.

## Abbreviations

PMNs: Polymorphonuclear leukocytes
ROS: Reactive oxygen species
MMPs: Matrix metalloproteinases
RANKL: Receptor activator of nuclear factor kappa beta ligand
OPG: Osteoprotegerin
PI: Plaque Index
GI: Gingival Index
PD: Probing depth
CAL: Clinical attachment level
TAC: Total antioxidative capacity
GCF: Gingival crevicular fluid
H_2_O_2_: Hydrogen peroxide
TMB: Tetramethylbenzidine
ELISA: Enzyme-linked immunosorbent assay
SD: Standard deviation.

## Additional Points

Clinical relevance is as follows:

- Scientific rationale: melatonin has a validated immunomodulatory and powerful antioxidant function. Its usage was considered as a novel addition to the standard of care procedures for periodontal therapy. The current study was conducted to evaluate the effect of topical melatonin gel as an adjunct to nonsurgical periodontal therapy.
- Principal findings: the local application of melatonin resulted in statistical significant reduction of probing depth and MMP-9 levels, while statistical significant increases the attachment level as well as total antioxidant capacity.
- Practical implications: the intrapocket local application of melatonin is highly recommended as an adjunctive to nonsurgical periodontal therapy.
